# Sharing and caring: Testosterone, fathering, and generosity among BaYaka foragers of the Congo Basin

**DOI:** 10.1038/s41598-020-70958-3

**Published:** 2020-09-22

**Authors:** Lee T. Gettler, Sheina Lew-Levy, Mallika S. Sarma, Valchy Miegakanda, Adam H. Boyette

**Affiliations:** 1 Department of Anthropology, University of Notre Dame, 244 Corbett Family Hall, Notre Dame, IN 46556 USA; 2 Eck Institute for Global Health, University of Notre Dame, Notre Dame, IN USA; 3 William J. Shaw Center for Children and Families, University of Notre Dame, South Bend, IN USA; 4 Department of Psychology, Simon Fraser University, Burnaby, Canada; 5 Department of Archaeology and Heritage Studies, Aarhus University, Aarhus, Denmark; 6 Institut National de Santé Publique, Brazzaville, Republic of the Congo; 7 Department of Human Behavior, Ecology and Culture, Max Planck Institute for Evolutionary Anthropology, Leipzig, Germany

**Keywords:** Evolution, Anthropology, Biological anthropology, Physiology

## Abstract

Humans are rare among mammals in exhibiting paternal care and the capacity for broad hyper-cooperation, which were likely critical to the evolutionary emergence of human life history. In humans and other species, testosterone is often a mediator of life history trade-offs between mating/competition and parenting. There is also evidence that lower testosterone men may often engage in greater prosocial behavior compared to higher testosterone men. Given the evolutionary importance of paternal care and heightened cooperation to human life history, human fathers’ testosterone may be linked to these two behavioral domains, but they have not been studied together. We conducted research among highly egalitarian Congolese BaYaka foragers and compared them with their more hierarchical Bondongo fisher-farmer neighbors. Testing whether BaYaka men’s testosterone was linked to locally-valued fathering roles, we found that fathers who were seen as better community sharers had lower testosterone than less generous men. BaYaka fathers who were better providers also tended to have lower testosterone. In both BaYaka and Bondongo communities, men in marriages with greater conflict had higher testosterone. The current findings from BaYaka fathers point to testosterone as a psychobiological correlate of cooperative behavior under ecological conditions with evolutionarily-relevant features in which mutual aid and sharing of resources help ensure survival and community health.

## Introduction

Among vertebrates that have evolved biparental care, testosterone (T) often serves as a physiological mediator of life history trade-offs^1–3^. In particular, males’ T is often higher during periods in which they compete with other males for mating opportunities and for resources (e.g. territory) that help them attract mates. In contrast, during periods when males partner with females to raise young, their T often declines, which helps divert limited time and energetic resources towards that cooperative parenting effort and away from competition^3–6^. Consequently, in such species, T has the potential to shape variation between males in health, survival, and reproductive fitness. Aligning with this general framework, in humans and other species, higher T has been linked to competitive, dominant behaviors related to pursuit of social status^5–8^. Meanwhile, there is also evidence that men with lower T may engage in greater prosocial, generous, and empathetic behavior^9–12^. Although the emergence of paternal care and heightened cooperation, respectively, were likely important to human evolutionary success, the links between human fathers’ T and these two behavioral domains have not been studied together, to our knowledge^9,13–16^.

Given the potentially critical roles for T in partnering and parenting, an increasing number of studies have explored variation in T between fathers and how it correlates to men’s roles within family systems, particularly their engagement in nurturant direct care^1,5,6,17,18^. With some notable exceptions^19,20^, the work that has linked lower T in fathers to greater paternal care has been conducted in the U.S., Europe, and the Philippines, which are cultural contexts in which care within the nuclear family has long been primarily prioritized or is increasingly emphasized (Philippines)^21–25^. In these societies, communal caregiving (or “cooperative breeding”), which is widely recognized as a critical adaptation that helped facilitate the evolution of human’s “slow” life history, is variably practiced^26–29^.

This stands in contrast to extensive cooperation in provisioning and direct care of children in certain other cultures. In particular, societies that still subsist (in part) via foraging often have norms of egalitarianism and cooperation that involve pooling of both time and energetic resources to help raise children^26,30–32^. While fathering and men’s contribution of resources to families and communities have been studied in a number of these societies^30–35^, relatively little is known about how men’s T relates to their roles as fathers in this type of hyper-cooperative, egalitarian setting^36–39^.

In contemporary foraging societies, fathers often spend meaningful daily time in close proximity to children and contribute to direct caregiving, particularly in comparison to men in small-scale societies that primarily subsist through other means (e.g. pastoralism; agriculture)^30,33,40,41^. The Congolese BaYaka foragers who are the focus of the present analyses are culturally and linguistically closely related to the more widely studied Aka foragers, who reside elsewhere in the Congo Basin^30^. In forest camps, Aka fathers have been shown to frequently hold their infants (22% of daytime hours) and are in close proximity to them (50% of a 24-h day)^33^. Similarly, across settings, BaYaka fathers often engage in direct caregiving and are commonly nearby and available to children^42^, though father-child interaction is higher in the forest than in village settings (see Methods). BaYaka fathers also cosleep with their families, facilitating nighttime father-child proximity^43^. Compared to those parenting behaviors, BaYaka men, like many forager fathers elsewhere, also typically devote substantially more daytime hours to subsistence activities, such as hunting/fishing and collecting honey, to acquire resources for their families and for sharing in the broader community^30,33,35,44^. In some otherwise egalitarian settings, hunting ability/reputation is a pathway through which men gain social status, which has been linked to greater fitness^45^. BaYaka men also acquire higher status via hunting success, although this is potentially attenuated in contemporary communities following reductions in elephant hunting. Moreover, BaYaka communities are similar to other mobile forager societies in making efforts to reduce status differentiation (i.e. they practice prestige avoidance)^33,46^. Importantly, at this study site, men’s reputations as providers and resource sharers are strongly positively linked^30^. From a social neuroendocrine perspective, we argue that lower T men may engage in greater sharing of resources with kin and other community members with whom they share reciprocally supportive relationships, especially when there are residing in the village (see Methods)^9,47^. Along those same lines, reduced T could be linked to men’s provisioning behaviors in this setting, given that many of those acquired resources will ultimately be pooled for and shared with the community, including targeted sharing with kin in the village setting^9,46^. Moreover, BaYaka fathers are also valued for their direct care and for their maintenance of positive marital functioning, which we would predict would be linked to lower T based on existing theoretical frameworks and past empirical research^6,17,48–50^.

To help address these questions regarding fathers’ T and roles within families and communities in an egalitarian setting, we drew on data from BaYaka foragers (n = 29) residing in northern Republic of the Congo^30^. The BaYaka are highly egalitarian and cooperative, pooling resources and sharing in care of children. In the forest, they subsist largely through hunting/fishing, collecting honey, and gathering plant resources. They also cultivate gardens, though less intensively than their farmer neighbors^30^. We specifically tested whether BaYaka fathers who were seen as better teachers (a form of direct caregiving), sharers of resources, providers, and husbands (avoiding marital conflict), respectively, had lower T compared to men who were seen as less effective in each of those domains. We then conducted analyses comparing predictors of men’s T between BaYaka and men from the neighboring Bondongo fishing-farming society (n = 16). Bondongo society is comparatively patriarchal and hierarchical with different values and models of family life than BaYaka^50,51^. Thus, to explore how cultural differences in hierarchy, cooperation, and family life may shape the relationships between men’s T and their roles as fathers we conducted pooled analyses for dimensions of fathering that were valued in both contexts (provisioning and reducing marital conflict). We predicted that T would be more strongly positively correlated to men’s rankings as providers among Bondongo men, given the status hierarchy in their community, compared to the more egalitarian BaYaka men. Similarly, we also tested whether there was a stronger positive relationship between T and marital conflict among Bondongo fathers compared to patterns for BaYaka men.

## Results

### Descriptive statistics and bivariate correlations

BaYaka men in the present study were 37.9 years of age (± 12.9 SD), on average, and their number of dependent children ranged from 1–8 (mean: 3.7 ± 2.2). In comparison, Bondongo men were of a similar average age (*p* > 0.9; mean: 37.2 ± 8.2) but had significantly more dependent children (*p* = 0.01; mean: 6.1 ± 3.7), with a range of 1–15 dependents, including a small number of families in which men had two wives and a large number of dependents^50^. On average, Bondongo men (mean: 8.8 mm ± 4.4) were also in better energetic condition, as measured via triceps skinfold thickness, relative to BaYaka men (*p* < 0.001; mean: 5.7 ± 0.9). BaYaka men’s T (averaged across samples; mean 67.9 pg/ml ± 28.4) was modestly but non-significantly lower than Bondongo males’ T (*p* > 0.2; mean 78.2 ± 31.2). See Table 1 for a full characterization of relevant descriptive statistics for each population.

**Table 1:** Descriptive statistics (n = 45).

	BaYaka (n = 29)		Bondongo (n = 16)		*p* value^a^
	Mean	*SD*	Mean	*SD*	
Age (years)	37.86	12.91	37.19	8.15	0.851
Number of dependent children	3.72	2.17	6.06	3.68	0.010
Triceps skinfold thickness (mm)	5.71	0.92	8.81	4.38	0.001
Testosterone (pg/ml)	67.92	28.36	78.19	31.15	0.267
Fathers’ Share ranking	5.72	2.22	–	–	–
Fathers’ Teach ranking	4.83	1.79	–	–	–
Fathers’ Provider ranking	6.94	1.90	8.45	3.67	–
Fathers’ Dispute ranking	3.12	0.96	3.11	1.35	–

^a^*p* values from unpaired Student’s t-tests. Men’s scores on the fathering domains reflect within-community rankings from peers and were consequently not compared in between-group comparisons.

In bivariate correlations, BaYaka men’s fathering rankings for Share, Teach, and Provider were all highly positively correlated (all Spearman’s *Rho* > 0.8, *p* < 0.0001). In contrast, BaYaka men’s scores in those fathering domains were not meaningfully or significantly correlated to their rankings for marital conflict (Dispute; all *Rho* < 0.1, *p* > 0.7). Men who were seen as better sharers, teachers, and providers had lower T (all *Rho* < -0.4, *p* < 0.05). Older men had lower T (*Rho* = -0.47, *p* ≤ 0.01) and were ranked higher for Share, Teach, and Provider (all *Rho* > 0.7, *p* < 0.0001). Those who were in better energetic condition tended to have higher T (Spearman’s *Rho* = 0.35, *p* = 0.06), but men’s rankings as fathers, including for Provider and Share, were not significantly correlated to their energetic status (all *Rho* < -0.1; *p* > 0.2). See Table 2 for a full summary of bivariate correlations for relevant study variables from BaYaka men.

**Table 2:** Bivariate correlations (Spearman’s *Rho*) between BaYaka sociodemographics, anthropometrics, and fathers’ rankings for family roles (n = 29).

	1	2	3	4	5	6	7	8
1. Age	1.0							
2. Number of children	0.65***	1.0						
3. Triceps skinfold thickness	− 0.22	− 0.28	1.0					
4. Log testosterone	− 0.47**	− 0.27	0.35	1.0				
5. Share ranking	0.78***	0.70***	− 0.14	− 0.58***	1.0			
6. Teach ranking	0.80***	0.69***	− 0.22	− 0.49**	0.88***	1.0		
7. Provider ranking	0.72***	0.71***	− 0.14	− 0.41*	0.87***	0.81***	1.0	
8. Dispute ranking	0.16	0.24	− 0.17	0.30	− 0.06	0.05	0.06	1.0

^*p* < 0.1; *p ≤ 0.05; **p ≤ 0.01; ***p < 0.001.

We have previously reported analogous correlative analyses from Bondongo men^43,44^ and include only a brief summary of key relationships here. In bivariate correlations, Bondongo men’s T was not significantly associated with their age, energetic condition, or rankings for Provider or Dispute (all *p* > 0.1). Older Bondongo men were ranked as better providers (*Rho* = 0.51, *p* < 0.05) but their age was not significantly correlated to marital conflict (*p* > 0.8). Bondongo fathers’ scores for Provider and Dispute were not significantly associated (*p* > 0.5).

### BaYaka fathering rankings and T

**Figure 1:**
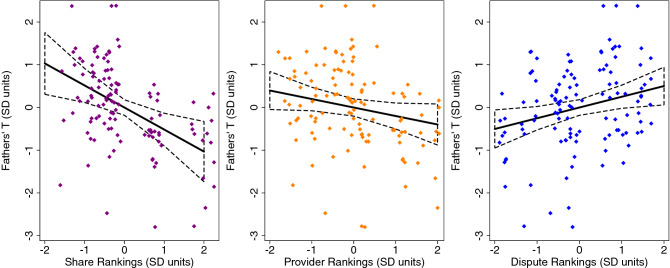
Linear plots (with 95% CI) of BaYaka fathers’ rankings for Share, Provider, and Dispute predicting their salivary T. All of the variables are in SD units. The linear plots were derived using predictive margins following the OLS regression models in Table 3. Scatter plots of the standard scores for men’s T in relationship to their rankings are overlaid.

In OLS regression models with clustered standard errors and adjusted for key demographic and energetic covariates, we found that BaYaka fathers who were ranked as better sharers within the community had lower T than men seen as poorer sharers (*p* = 0.005; Table 3; Fig. 1). Similarly, men ranked as disputing less with their wives had lower T than men in more conflicted marriages (*p* = 0.017; Table 3; Fig. 1). Men who were ranked as better providers tended to have lower T, but the 95% CI included zero (CI: -0.42, 0.01; *p* = 0.061) and the effect size (standardized B = −0.20) was comparatively smaller than for the other ranking domains, particulary for Share (B = −0.52). With the inclusion of covariates, there was not a significant relationship between men’s rankings as teachers and their T (*p* > 0.1; Table 3). BaYaka men in better energetic condition had higher T compared to more energetically stressed men (all *p* < 0.05). Older men generally had lower T than younger men (all *p* < 0.1), except in the “Share” model (*p* > 0.5). See Table 3 and Fig. 1 for the full results of these models and visual representations of the patterns. In these analyses, the VIFs for the independent variables were less than: 2.0 (Dispute model), 3.0 (Work model), or 4.0 (Teach and Share models).

**Table 3:** OLS regression models predicting BaYaka fathers’ testosterone from their rankings for sharing, provisioning, teaching, and marital conflict (n = 29).

	Model 1: share			Model 2: provider		
	*b*	*95% CI*	*p*	*b*	*95% CI*	*p*
Share^a^	− 0.52	(− 0.87, − 0.17)	0.005			
Provider^a^				− 0.20	(− 0.41, 0.01)	0.061
Age (years)	− 0.01	(− 0.03, 0.01)	0.547	− 0.03	(− 0.04, − 0.01)	0.002
Triceps skinfolds (mm)	0.21	(0.03, 0.39)	0.025	0.25	(0.08, 0.42)	0.006
Total # of children	0.12	(0.02, 0.22)	0.025	0.12	(0.01, 0.23)	0.038
Model R2		0.29			0.24	

	Model 3: teach			Model 4: dispute		
	*b*	95% CI	*p*	*b*	95% CI	*p*
Teach^a^	− 0.24	(− 0.56, 0.08)	0.132			
Dispute^a^				0.25	(0.05, 0.45)	0.017
Age (years)	− 0.02	(− 0.04, 0.00)	0.071	− 0.03	(− 0.04, − 0.01)	0.004
Triceps skinfolds (mm)	0.22	(0.01, 0.43)	0.039	0.24	(0.07, 0.41)	0.009
Total # of children	0.09	(− 0.02, 0.19)	0.113	0.03	(− 0.07, 0.13)	0.589
Model R2		0.24			0.28	

Results reflect analyses of n = 117 testosterone data points from 29 men. OLS models include standard errors clustered by individual father to account for repeated sampling of testosterone.

^a^Share: fathers' peer ranking scores for sharing of resources in the community. Provider: fathers' peer ranking scores for provisioning of resources; Direct: fathers' peer ranking scores for teaching their children; Dispute: fathers' peer ranking scores for marital conflict. All ranking scores have been converted to standard deviation units (z-scores).

### BaYaka and Bondongo fathering and T

**Figure 2:**
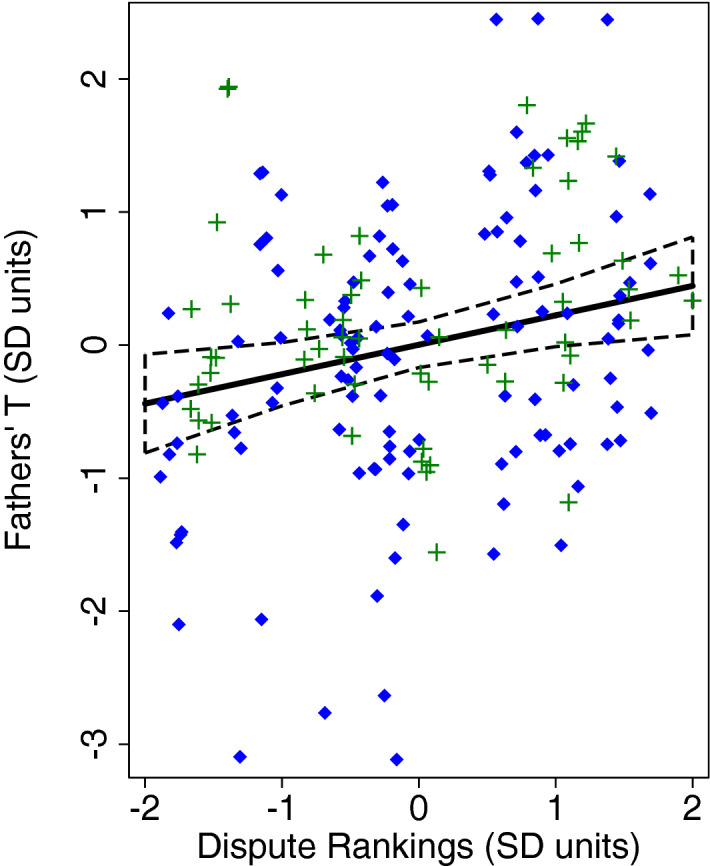
Linear plot (with 95% CI) of BaYaka and Bondongo fathers’ rankings for Dispute predicting their salivary T. Both of the variables are in SD units. The linear plots were derived using predictive margins following the OLS regression (model 2) in Table 4. Scatter plots of the standard scores for men’s T in relationship to their rankings are overlaid. Blue diamonds: BaYaka data. Green crosses: Bondongo data.

We then ran analyses that included both BaYaka and Bondongo fathers to test whether ethnicity moderated the relationships between similar local dimensions of fathering (Provider, Dispute) and T. For marital conflict, we did not find a significant interaction (Ethnicity × Dispute; *p* > 0.6). However, there was a main effect of T, as men (in both societies) with higher T were ranked as having greater conflict with their wives (*p* = 0.013). Because that significant effect is conditional to the interaction term, we ran a complementary model without the interaction. In this model, we observed an analogous pattern linking higher T to greater conflict, with a similar effect size (*p* = 0.009; Table 4; Fig. 2). Finally, we found that the slope of the relationship between T and men’s Provider rankings was positive for Bondongo men and modestly negative for BaYaka men, but the interaction term (Ethnicity × Provider; *p* = 0.111) was not significant. Across these models, older men had lower T than younger men (all *p* < 0.001). BaYaka and Bondongo men did not significantly differ for T in any model (all *p* > 0.6), paralleling the non-significant bivariate comparison in Table 1. See Table 4 and Fig. 2 for the full results of these models and visual representation of the pattern for Dispute. In these analyses, the VIFs for the independent variables were less than: 2.0 (Dispute model) or 3.0 (Provider model).

**Table 4:** OLS regression models predicting BaYaka and Bondongo fathers’ testosterone from their rankings for provisioning and marital conflict (n = 45).

	*b*	*95% CI*	*p*				
**Model for Provider**							
*Main effects*							
Provider^b^	− 0.09	(− 0.36, 0.18)	0.503				
Ethnicity	0.13	(− 0.35, 0.61)	0.600				
Age (years)	− 0.04	(− 0.05, − 0.02)	0.0001				
Triceps skinfolds (mm)	0.01	(− 0.05, 0.06)	0.807				
Total # of children	0.04	(− 0.02, 0.10)	0.170				
*Interaction term*							
Ethnicity × Provider	0.24	(− 0.06, 0.54)	0.113				
Model R2		0.21					

	Model 1			Model 2			
	*b*	*95% CI*	*p*		*b*	*95% CI*	*p*
**Model for Dispute**							
*Main effects*				*Main effects*			
Dispute^b^	0.25	(0.05, 0.44)	0.013	Dispute^b^	0.22	(0.06, 0.38)	0.009
Ethnicity	0.10	(− 0.36, 0.57)	0.661	Ethnicity	0.10	(− 0.36, 0.57)	0.662
Age (years)	− 0.03	(− 0.05, − 0.02)	0.0001	Age (years)	− 0.04	(− 0.05, − 0.02)	0.0001
Triceps skinfolds (mm)	0.02	(− 0.03, 0.07)	0.412	Triceps skinfolds (mm)	0.02	(− 0.03, 0.07)	0.393
Total # of children	0.03	(− 0.03, 0.09)	0.338	Total # of children	0.03	(− 0.03, 0.09)	0.348
*Interaction term*							
Ethnicity × Dispute	− 0.07	(− 0.40, 0.27)	0.692				
Model R2		0.25				0.25	

Results reflect analyses of n = 177 testosterone data points from 45 men (29 = BaYaka). OLS models include standard errors clustered by individual father to account for repeated sampling of testosterone. Ethnicity: 0 = BaYaka; 1 = Bondongo.

^a^Provider: fathers' peer ranking scores for provisioning of resources; Dispute: fathers' peer ranking scores for marital conflict. All ranking scores have been converted to standard deviation units (z-scores).

## Discussion

A major goal of these analyses was to test for relationships between T and locally-defined measures of fathering quality in a small, egalitarian society. There has been limited past research on the biology of fatherhood in forager societies in which men are often committed to caring for their immediate families but are also contributors to the broader community’s pooled resources and shared engagement in collective caregiving^36,38,39,52^. We found that BaYaka fathers who were seen as better community sharers and those in less conflicted marriages, respectively, had lower T than their peers. Below, we contextualize our findings for BaYaka fathers and comparisons with their fisher-farmer Bondongo neighbors within theoretical frameworks and empirical research related to the psychobiology of family life, cooperative behavior, and competition/risk taking.

### BaYaka fathering rankings and T

Consistent with our predictions, we found that BaYaka fathers who were ranked by their peers as being more generous sharers with the broader community had lower T than men who were seen as poorer sharers. Gettler has argued that one potential evolutionary and psychobiological implication of some men experiencing declines in T when they became committed fathers is that this might enhance their ability to cultivate social capital within their communities through greater cooperative and generous behavior^9^. This proposed neuroendocrine role of reduced paternal T fits within broader conceptual models that emphasize the importance of cooperation, reciprocal altruism, and empathy to the evolution of human life history^13,14,26,32^. Our finding for BaYaka fathers’ sharing is consistent with this framework. This result also had a meaningful, medium-level effect size (standardized B = -0.52) that well exceeds the effect sizes from recent meta-analyses on human T and fathering and past meta-analyses examining the positive associations between T, competition, and dominance behaviors^7,53,54^. To our knowledge no other research has specifically explored the relationship between fathers’ T and cooperative behavior, beyond nuclear family childcare roles^9^.

However, relevant research outside of family life has found that men with lower T commonly show greater generosity and empathic capacities^10–12^. Generally consistent with these patterns, a small number of recent studies have also found that lower T individuals have more social support, feel socially closer to others, and generate new friendships within social networks^55–57^. In contrast, experimental studies involving T administration have shown that men with elevated T will also circumstantially behave generously if such behavior helps increase social status^58,59^. In BaYaka communities, men are viewed positively for their generosity and likely gain benefits from community sharing (e.g. through reciprocity)^60,61^. Yet, in this egalitarian setting there are strong cultural values and practices geared towards reducing status hierarchy (see Methods)^30,33,62^. Thus, we suggest that our finding linking lower T and better sharing for BaYaka men aligns with social dynamics within their cultural context as well as past psychobiological research on T and generosity.

In our full models, BaYaka men who were ranked as better providers also tended to have lower T than men who were seen as poorer providers. This finding was not statistically significant and the effect was smaller (standardized B = -0.21), compared to the results for Share. This may reflect the strong positive relationship between men’s scores for Share and Provider and is potentially consistent with the idea that community members are basing BaYaka men’s rankings as providers at least partially on men’s sharing of acquired resources^63^. In addition, in multiple societies that still engage in foraging as a part of their routine subsistence, men who are better hunters often achieve some semblance of higher social status, despite egalitarian social norms, and hunting ability and reputation have been linked to higher fitness^45^. These competition- and status-related aspects of hunting in such societies could theoretically be linked to elevated T^6,7,16^. Indeed, among Amerindian Tsimane forager-horticulturalists men who achieved kills during hunts showed short-term increases in T, potentially reflecting these status-competition psychobiological effects^37^.

Meanwhile, in BaYaka communities, men who were skilled, successful elephant hunters (*ntuma*) are remembered historically as having had higher social standing in their communities. However, there were no *ntuma* in the community at the time of our data collection. Elephant hunting is now illegal and severely reduced in practice today and the importance of men’s hunting as a pathway to status is potentially attenuated in contemporary BaYaka communities. In comparison, local healers (*nganga*) and BaYaka council members remain prestigious positions within the contemporary community, and help foster cooperation through ceremony and conflict resolution^64,65^. Finally, we note that hunting is only one component of men’s provisioning in this setting, and our Provider ranking captures this broader breadth of subsistence activities, according to the participants’ characterizations^30^. Collectively, these issues help to highlight the relative dearth of studies on social neuroendocrine function among contemporary forager societies, limiting our ability to disentangle the importance of variation in status, cooperation and generosity, and paternal care in different societies^36,38,39,52^.

We also did not find a significant relationship between BaYaka men’s rankings as teachers and their T after adjustment for covariates. BaYaka fathers’ teaching involves direct interaction using a variety of behaviors, such as instruction and opportunity scaffolding^66–68^. Fathers have also been observed to do more teaching with very young children, whereas less of this “vertical transmission” from parents occurs for older children^67,69^. As direct caregivers, BaYaka fathers are often warm, nurturing, and patient^33^. This parenting style may be beneficial during teaching of young children and this type of nurturant direct care, especially with young children, is likely to be linked to lower paternal T, based on theory and past research elsewhere^1,6,17,18^. Thus the lack of a significant association and the relatively smaller effect size (standardized B = − 0.24) between men’s T and their teaching ran somewhat counter to our predictions. We do note that while teaching emerged as a locally-valued domain of fathering and may be significant for children developmentally^70^, BaYaka fathers may spend relatively less time engaged in teaching of their offspring, compared to other direct paternal care. For example, while Aka fathers were the second most important adult teachers of infants, their contribution was still much lower than those of mothers (12% vs. 59% of observed teaching). However, fathers’ teaching nearly exceeded all other adults combined (15%)^66^. Recent work by Lew-Levy and colleagues has shown that older BaYaka children and adolescents commonly accompany adults (not necessarily their parents) to forage^71^, and those trips afford opportunities for learning and teaching^67,69^. In future work, we hope to include further observational data on specific domains of men’s daytime direct care as well as family cosleeping, as these have been linked to lower or declining paternal T in multiple other settings^20,22–25,72,73^.

### BaYaka and Bondongo fathering and T

Among BaYaka and Bondongo participants, there was a consistent emphasis that “good” fathers work to reduce conflict with their wives, as negative interactions between parents can lead to poor outcomes for children^30,51,74^. Despite this similar recognition of the importance of positive marital functioning in both societies, the two cultures have differing perceptions of the place of spousal conflict in day-to-day life, which align with broader cultural values in each community. Specifically, BaYaka are relatively gender egalitarian and value individual autonomy. They disapprove of conflict between wives and husbands and particularly have cultural mechanisms for attenuating and avoiding control of one partner over the other. Meanwhile, Bondongo communities are more hierarchical and patriarchal, thus men are generally higher in status and power than women, and disputes are seen as a part of normal day-to-day marital functioning^30,51,74^.

Based on these cultural differences, we predicted that Bondongo marital conflict would be more strongly related to men’s T than among BaYaka fathers. This prediction was not supported. Rather, we found a main effect for Dispute, such that men in both societies who were ranked as having more marital conflict with their spouses had higher T than men in more harmonious relationships. We also note that in the Dispute model focusing solely on BaYaka men we observed a complementary, stand alone finding relating greater marital conflict to higher T. These main effects potentially reflect that cultural variation in norms regarding the acceptability of marital conflict may have little effect on the actual frequency of such conflict. Our findings also align with findings elsewhere from a range of socio-ecological settings. For example, U.S. men with higher T reported that they felt less satisfied and committed to their relationships and had more marital conflict^49,75^, and in a large decade-long longitudinal U.S. study higher T men had greater risk of divorce^48^. Similarly, in a large longitudinal analysis from the Philippines, men with greater T functioning were more likely to experience relationship dissolution over a five-year period^76^. It is somewhat difficult to compare effect sizes across these varied studies. However, the bivariate correlation between marital conflict and men’s T in our combined sample (*r* = 0.33) is similar in size to the findings from Edelstein and colleagues’ (2014) research linking higher T to lower relationship satisfaction, investment, and commitment in the U.S. (*r*s = -0.29 to -0.36)^49^. In total, our findings add additional cross-cultural support to a growing body of literature linking higher T to poorer relationship functioning and outcomes in very different societies.

Finally, we did not find that the relationship between men’s Provider scores and T were significantly different between the two cultural groups. However, we do note that the slope of the lines relating men’s Provider rankings and T were in the opposite direction for men in the two communities (Bondongo: positive; BaYaka: negative). This reflects our past findings showing that Bondongo fathers who were rated as better providers had higher T than their peers^77^, and an opposite non-significant pattern linking lower T to higher Provider scores for BaYaka fathers in the present analyses. These patterns hint at the potential importance of cultural variation in social norms and complements foundational^36^ and recent^39^ anthropological work that similarly explored cross-cultural variation in fathers’ T.

Muller et al. (2009) found that Hadza forager fathers in Tanzania had lower T than non-fathers while among their Datoga pastoralist neighbors there was no significant difference for T between fathers and non-fathers. Building on this work, Alvarado et al. (2019) compared Hadza and Datoga men with Qom transitional foragers of Argentina. Relative to young Datoga fathers, Hadza and Qom men with children had lower T during their reproductive primes. The authors suggested that Datoga men’s elevated T as young fathers is particularly linked to the cultural practice of polygyny (i.e. involving competition) and the relative lack of routine contact between men and their families, due to men’s subsistence. Meanwhile, Qom and Hadza fathers more routinely engage in proximate interactions with their families and are generally serially monogamous^39^. In the present study, we need to be restrained in over-interpreting non-significant results. Yet, along this past research and other relevant work^19,20,78^, we hope our findings can help bring further attention to the potential importance of operationalizing cultural norms and practices in studies of social neuroendocrine function.

### Limitations

There are limitations to the present study that merit attention. As we have discussed in past work from this study, our sample sizes of fathers were relatively small compared to some studies of paternal psychobiology in industrialized settings in more highly populated societies^72,79–82^. However, we also note that our pooled analyses (n = 45) and BaYaka sample size (n = 29) compare favorably to other recent work in this area^25,72^, especially research in similar societies^39^. That said, small sample sizes limit statistical power, as may have been the case in our moderation analyses predicting T from men’s provider rankings (Provider × ethnicity), and can also contribute to inflated effect sizes for statistically significant results^83^. While there has been substantial growth in the study of paternal psychobiology, much of the emerging research is in the U.S., Europe, and similar settings^53,54^. The present study makes an important, complementary contribution by focusing on these questions in two small-scale, subsistence-level societies, which differ politically, economically, and culturally from one another and from most prior study samples in this research area^17^. In that vein, as we have discussed in our past work from this site, smaller sample sizes are a research design trade-off that result from working at a highly remote field site with participants residing in small communities^77^. To that end, for each of the two communities, our sample of fathers represents ~ 90–100% of the eligible men in the village at the time of data collection. Moreover, to help attenuate sample size concerns, we collected repeated samples across participants and also used data analytical techniques that maximized the information from these repeated observations.

In addition, the BaYaka do not record their calendar ages, thus we estimated an approximate age based on a procedure from Diekmann and colleagues and with their assistance^84^. In validating their method, Diekmann et al. found the calculations were reliable within a year of known ages (median: 4 months; mean: 11 months) for another forager society, giving us confidence in the BaYaka calculated ages^84^. Still, because of inter-correlations between BaYaka age, T, and fathering rankings, the reliability of these age calculations could be potentially concerning in terms of adjusting our models for age. For BaYaka men, their calculated ages and T were qualitatively more strongly correlated (*Rho* = -0.46) than for the Bondongo (*Rho* = -0.34), who do record their ages. While populations can vary in age-related declines in T^39,85^, we suggest this is one helpful indicator of the validity of the calculated ages for BaYaka men. Moreover, the BaYaka age-T-rankings inter-correlations could pose potential issues for multi-collinearity for the independent variables in our regression models. Following each of our regression models, we calculated variance inflation factors (VIF) for the predictors. Although conventions can vary, it is common to use VIF of > 10 as an indicator of concerning multi-collinearity^86^, and the calculated values for the present analyses were well below this threshold, as we reported in the Results.

Finally, there was a minor unintended difference in the handling of the saliva samples our team collected from the participants in the two communities. During both field seasons (see Methods), we froze the saliva samples on site in portable liquid nitrogen dewars, and they remained frozen until shipment. The Bondongo samples were kept frozen throughout their transport to the U.S. During shipment of the BaYaka samples, we encountered a logistical problem, which resulted in the saliva samples going through a freeze–thaw cycle while in transit. All the BaYaka samples were exposed to identical conditions, and salivary T is generally robust to limited freeze–thaw cycles and short-term exposure to ambient temperature^87,88^, which attenuates concerns over this issue.

## Conclusion

We found that BaYaka fathers in a highly egalitarian contemporary forager society had lower T if they were considered better community sharers, compared to men seen as less generous. Selection for paternal care and the capacity for hyper-cooperation were likely critical to the evolutionary emergence and success of human life history^13–15,26,32^. Thus, our results are relevant to how we conceptualize the physiological mechanisms that might help underpin (or respond to) the facultative expression of these evolutionarily important suites of behavior^9,16^. These findings specifically point to T as a component of prosocial psychobiology in an ecology with evolutionarily-relevant conditions in which extensive cooperation helps ensure survival and community health. We also showed that in two neighboring small-scale societies men with higher T were similarly viewed as having more conflict with their wives despite cultural differences for norms around egalitarianism/hierarchy and family life. Collectively, our results highlight the ongoing need for studies of human biology to rigorously operationalize the role of culture in explaining biological diversity and in contextualizing its commonalities for critical aspects of human experience, such as the psychobiology of family life.

## Methods

### Study populations

As part of a broader project focusing on fathering, family function, and child well-being, our research team collected data from Bondongo men (n = 16) in 2016 and BaYaka men (n = 29) in 2017 in Likouala department (province) in a remote part of northern Republic of the Congo. Initial permission to conduct research in the village was given by the village council during a community meeting with AHB in 2015. These communities are three to six days (via truck and motorboat) from Brazzaville, the capital of the Republic of the Congo. Fathers were eligible to participate in the study if they had at least one child who was less than 18 years old; all participating men had at least one biological child that met this age criterion. Data collections included demographic, anthropometric (triceps skinfold thickness), and salivary biomarker data, as well as family role ranking measures for fathers. We collected triceps skinfold thickness data triceps using Lange skinfold calipers by standard techniques. Because the BaYaka do not record age, we estimated an approximate age following methods and with assistance from Diekmann and colleagues^84^. The Bondongo record and know their ages based on calendar dates of birth and reported them as such. Because our analyses draw on similar data from prior research from this site, our methods are comparable to those we have reported previously^30,50,51^.

The study village is home to ~ 400 people, with around half the population being BaYaka and the other half Bondongo. The two communities live in ethnically segregated neighborhoods. In the BaYaka section of the village, there are roughly five family “hamlets” that are organized fluidly by kinship. In the Bondongo neighborhood, membership in patrilineal clans shapes the organization of families and buildings, with each individual household having its own compound with a dwelling house and kitchen area. In the following paragraphs, we include further cultural details about each community that are relevant to the present analyses. Following prior comparative research from this region, we refer to the BaYaka as “foragers” and the Bondongo as “fisher-farmers” throughout this article^89,90^. We recognize that these terms gloss over complex histories and identities^91^, and we also report details of their subsistence strategies below^30,51^.

### BaYaka foragers

BaYaka culture comprises strong values of egalitarianism, cooperation, sharing, and respect for the autonomy of all individuals. Within families, men and women often work collaboratively on foraging and domestic activities, and equitable marital relationships and the avoidance of routine conflict are culturally valued. BaYaka society is also status-averse, and there are cultural practices in place to help mitigate hierarchy within the community^30,33,47^. In terms of daily subsistence, BaYaka individuals often leave the village to acquire resources in the forest and obtain a major part of their daily subsistence from wild forest foods. They characterize themselves as forest experts, and are viewed as such by the Bondongo. In the forest, they engage in hunting, fishing, and trapping and also collect honey and gathering plant resources. Hunting is primarily a male subsistence activity in this community. While BaYaka men’s hunting is valued, hunting success is not a strong source of status differentiation, and BaYaka communities attenuate status through practices such as prestige avoidance. For instance, people only speak of hunting accomplishments far after the fact. Any attempts men might make to garner public recognition of their catch would lead them to be the subjects of “rough-joking,” and they would be called stingy by anyone who has not received a share. In other words, in terms of social standing, men are generally better off silently sharing any resources they acquire^33^. Men also meaningfully contribute to subsistence through the other forest activities and those in the village. Specifically, BaYaka families also practice modest levels of swidden agriculture, planting and cultivating garden plots for crops such as cassava, though they tend their gardens much less intensively than Bondongo. Finally, in the village, Bondongo families often employ BaYaka adults for a number of domestic and subsistence activities, including to collect forest resources for both consumption and other needs (e.g. building materials). BaYaka individuals in this community often move their residence multiple times per year, including residing in the shared village with the Bondongo, but also relocating to forest camps for up to two month periods at lease twice per year^30,47^.

The BaYaka pool and share resources within the community, including hunted animal protein, honey, and collected and cultivated plants. Among recipients, meat is the most desired product to be shared, with plants being somewhat secondary. In the forest camps, all food is shared widely with those present, as desired and needed. Kinship is not a strong determinant of forest sharing^47^. In the village setting, kinship appears to be a stronger influence on resource sharing. This may be because of greater comparative population density and close proximity of kin, relative to forest camps, which tend to be smaller and more heterogeneous in terms of kinship^47^.

### Bondongo fisher-farmers

The Bondongo are a Bantu-speaking people who engage in swidden agriculture to cultivate crops such as cassava (manioc), plantains, and corn. Bondongo society is patriarchal and socially stratified and hierarchical based on gender, age, and both ascribed and acquired status^30,51^. In this community, there is a rigid sexual division of labor. After men clear the garden plot, agriculture is exclusively the work of women. Men make subsistence contributions through hunting parties, fishing using lines and traps, and cultivating palm wine, or *molenge*. As we have described previously^77^, Bondongo men’s subsistence activities are often dangerous, including involving risks of drowning while fishing, falling while scaling trees, or incurring harm while burning and clearing garden plots. Men’s subsistence roles, particularly their cultivation of *molenge*, are means by which they acquire status and maintain social relationships within the hierarchical community. Moreover, compared to women, men wield more overt political power and hold positions of authority in the village government, starting with the Precôt, or elected chief. Although men typically hold higher status than women, Bondongo husband-wife relationships are cooperative and economically productive, and wives share in decision-making power within marriages and families. Among Bondongo families, marital conflict often involves verbal aggression and (less commonly) physical aggression between husbands and wives and is seen as a part of normal marital relations^50,51^.

### Cultural models of fathering

A primary focus of this biocultural research was to use mixed methods to understand and model culturally defined roles for fathers in each of these societies. In separate seasons of fieldwork, AHB and SLL conducted qualitative ethnographic interviews with men and women of each community to define the relevant local cultural domains for the responsibilities of a father and the qualities that made a “good” father^30,51^. Within both communities, consistent themes emerged in these interviews, which we describe in further detail below. Once we established these domains for internal cultural perceptions of fatherhood, we then had fathers rank one another according to those characteristics and tested whether those rankings helped explain fathers’ psychobiology (see below).

### BaYaka fathering & rankings

BaYaka individuals characterized good fathers as those who: hunted, gathered honey, and collected and other forest resources for their families (Provider); did not fight with their wives (Dispute); welcomed others to the community and shared resources well within the community (Share); and taught their children to forage (Teach)^30^. For the ranking task, we recruited an opportunistic subsample of BaYaka fathers from the larger study (n = 21) to rank the other participating fathers on the cultural domains we extracted from the interviews. The sub-set of BaYaka fathers who provided the rankings (n = 21) did not significantly differ from the fathers who did not rank their peers but who had ranking data (n = 10) for any key study covariates (e.g. age, number of children, energetic condition), testosterone, or the rankings themselves (all *p* ≥ 0.3). The current analyses include 29 BaYaka fathers because two men did not have testosterone data. During the ranking task, we showed the participant a set of photographs of their peers and then asked them to place these in order, in piles, from “first” to “last” for each domain. We asked that fathers attempt to uniquely rank each of their peers but permitted ties. We recorded the last position as a score of “1” and the highest score as the total number of piles made. Using the ranking scores from the twenty BaYaka participants, we then calculated an average father “quality” peer-ranking score, from their fellow fathers, in each domain. The Cronbach’s alphas for these four peer-rankings ranged from 0.76–0.86^30^. For both the BaYaka and Bondongo (below) rankings, we did not collect data on subsistence returns to correlate to men’s rankings as providers or sharers of resources (BaYaka). In past work on forager men’s hunting reputations, skills, and returns, it has been argued that rankings from long-time residents of the same community (i.e. who are well known to each other), especially in kin networks, are likely to be reliable indicators of hunting success and ability^63,92^. BaYaka men tend to routinely focus on small- and medium-sized game when hunting, which have lower variance than larger game^93^. Moreover, the BaYaka Provider rankings are inclusive of men’s acquisition of other consistent resources, such as forest plants, collected through other means. This focus on lower-variance targets should increase the ability of community members to reliably rank BaYaka fathers in subsistence-related domains^63,92^. Finally, the BaYaka men in the study were generally related to one another, either genetically or through marriage, and knew each other well. Those born in the community had known each other throughout their lives. Meanwhile, men who had married into the community typically had local kin and, by the time they had children, were familiar to community members.

### Bondongo fathering and rankings

Bondongo participants described good fathers as those who: provided resources for their families (Provider); did not fight with their wives (Dispute); and engaged in direct care (Direct: e.g. shaping children’s proper socialization; caring for ill children). As we have described in prior work, fathers’ indirect care (Provider) was much more highly valued among the Bondongo, compared to direct caregiving (Direct), and Bondongo fathers engaged in relatively little direct care, on average^43,44^. In the current analyses we only included Bondongo rankings for Provider and Dispute, as they could be compared to similar domains among the BaYaka. In the Bondongo sample, all participating fathers in the current analyses (n = 16) ranked one another using the same procedure we outlined above for the BaYaka rankings. The Cronbach’s alphas for these peer-rankings were 0.84 (Dispute) and 0.95 (Provider)^44^. The Bondongo men in the sample knew each other well, as most had grown up together and/or were related through kinship by various degrees. Two men were relatively recent arrivals: one an immigrant who has lived there for almost 10 years and has a local wife and children; the other arrived more recently along with his wife and children. Both were well integrated into village life.

### Salivary T data

Adults provided 2 mL of saliva via passive drool in polypropylene tubes for up to five evenings. Over 82% of Bondongo men and 75% of BaYaka men provided 4–5 samples while the remaining men in each group gave 3 samples. In the present study, we analyzed 177 T data points from n = 45 men. For both communities, the collections began no earlier than 16:30 and generally concluded by 18:30. This represents the early evening period when daytime work concludes, and individuals return from the river, forest, and gardens, and shift their attention towards household tasks. We specifically designed our study to collect repeated evening samples from individuals because T’s well-characterized diurnal curve typically reaches its nadir by late afternoon and remains at that level through the evening, until sleep commences^94–96^. This design thus helped our small research team minimize between-subject and between-day variability in T that might be attributable to the time of sampling.

Samples were frozen on site at the field location in portable liquid nitrogen dewars and remained frozen until shipping to the University of Notre Dame (UND). Bondongo samples were kept frozen in a liquid nitrogen dry shipper during transport to UND. The BaYaka samples were shipped similarly, but due to logistical problems during shipment, the saliva samples went through a freeze–thaw cycle while in transit. Research on handling conditions and freeze–thaw cycles for saliva samples assayed for T has shown that this should not meaningfully impact T^87,88^. Upon arrival to UND, the samples were then stored at -80 C until we assayed them for T. Using commercially available kits, we analyzed the samples for T at UND’s Hormones, Health, and Human Behavior Lab (Salimetrics, Kit Number: 1-2402). The inter-assay coefficients of variation (CV) for the low and high controls were as follows: Bondongo T, 9.6% and 5.6%; BaYaka T, 19.0% and 11.2%. The intra-assay CV was 4.6%. In all relevant analyses, the values for T were natural log transformed to adjust for non-normal distribution of the data. We excluded three T data points (one each for two Bondongo men and one BaYaka man) based on T values that were 3+ SDs from the mean for the sample.

### Covariates

We included theoretically- and empirically-informed covariates that we predicted could confound the relationship between our core predictors (fathers’ rankings) and men’s T. We specifically adjusted for men’s age, energetic condition (triceps skinfold thickness), and number of dependent children. In both of the study populations, older men have lower T than younger men (Table 2)^77^, and men may also have the opportunity to build their reputations in fathering-related domains (e.g. provisioning) with age (Table 2)^32^. In some energetically-constrained settings, men in poorer energetic condition have lower T than their better nourished peers, including among BaYaka (Table 2). Finally, men who are higher quality fathers may have more surviving children, and in some settings having more, particularly younger, children may correlate with men’s T^21,78^.

### Statistical analyses

We conducted all statistical analyses in Stata v. 14.0 (Stata Corporation). We treated men’s T, fathering rankings, ages, number of children, and skinfold thickness as continuous variables. In reporting descriptive statistics in Table 1, we compared BaYaka and Bondongo men for key study variables using unpaired Student’s t-tests. Following our prior work^77^, we then used a series of OLS regression models with standard errors clustered by individual to account for the repeated sampling of T in order to test for links between men’s T and fathering rankings. Using this approach, we predicted BaYaka men’s T from their fathering rankings adjusting for covariates (men’s age, number of children, and skinfold thickness). We have previously reported complementary analyses for Bondongo men’s T and fathering rankings^50,77^.

We then ran models in which we aggregated and compared the BaYaka and Bondongo data. In these models predicting men’s T, we included an interaction term between a categorical variable (“Ethnicity”) that identified individuals as Bondongo or BaYaka and our key independent variables (continuous ranking scores for Provider and Dispute, respectively). As we described above, these are the two domains in which there were similarities between the two cultural groups in their descriptions of locally-valued fathering roles. We included the interaction terms between Ethnicity and the independent variables because we predicted that the slopes of the relationships between fathers’ T and rankings for Provider and Dispute, respectively, would be significantly different in the two groups. To facilitate comparisons and discussion of relative effect sizes across models, we converted men’s log-transformed T and father ranking scores to z-scores. Following the regression models, we used Stata’s “estat vif” command to calculate variance inflation factors (VIF) for the independent variables. We evaluated statistical significance at *p* < 0.05.

### Ethics

Initial permission to conduct research in the village was given by the village council during a community meeting with AHB in 2015. The Centre de Recherche et D'etudes en Sciences Sociales et Humaines granted permission to conduct the research in Republic of the Congo, and the Institutional Review Boards of Duke University (Protocol # 2017-0038) and the University of Notre Dame (# 18-02-4397) also approved the study. Individual informed verbal consent was obtained from all adult participants. The verbal consent process as well as all data collections and methods were conducted based on Duke University and University of Notre Dame ethics guidelines and approvals.

## Supplementary information

Supplementary information  1

Supplementary information 2

Supplementary information  3

## Data Availability

We have included the data necessary to replicate the patterns in this article in a .csv file that can be found in the Supplementary information. Because the BaYaka and Bondongo communities are small, men’s ages and their number of dependents represent identifiable information. Therefore, we took two steps to help protect their identities and privacy. First, we used a published R script for a package known as “synthpop” to generate synthetic data for age and number of dependents that approximate the real data ^97^. We then converted those two synthetic variables to z scores. Thus, we note that if one uses the provided data to replicate the analyses, the coefficients for the key predictors (i.e. fathers’ rankings) will be nearly identical, while the coefficients for age and number of dependents will differ, reflecting those synthetic variables being in standard scores. In the Supplementary information, we have included a brief codebook (in .doc format) explaining the variables in the .csv file and the statistical analyses in the manuscript. We have uploaded the data and the codebook to a public repository on GitHub (https://github.com/ahboyette/Gettler_etal_NatSciRep_public_files).
