# Supplementary material for: Sharing and caring: Testosterone, fathering, and generosity among BaYaka foragers of the Congo Basin
Source: Sci Rep. 2020 Sep 22;10:15422. doi: 10.1038/s41598-020-70958-3 (PMC7508877; doi:10.1038/s41598-020-70958-3)
Supplement: Supplementary file 1 — Supplementary information 1 [file 41598_2020_70958_MOESM1_ESM.docx]

**Sharing and caring: Testosterone, fathering, and generosity among BaYaka foragers of the Congo Basin**

Lee T. Gettler

Sheina Lew-Levy

Mallika S. Sarma

Valchy Miegakanda

Adam H. Boyette

*Supplemental materials

Gettler et al. Nature Scientific Reports codebook

*We note that we have also uploaded this codebook and the relevant study data to a public repository on GitHub: <https://github.com/ahboyette/Gettler_etal_NatSciRep_public_files>

id: participant identifier

ethnicity: 1==BaYaka; 2==Bondongo

agez_syn: synthetic variable for men’s ages following z-score transformation

childtotalz_syn: synthetic variable for men’s total number of dependent children following z-score transformation

sft_all: men’s triceps skinfold thickness

testo: men’s raw testosterone values (pg/ml)

logtesto: men’s natural log transformed testosterone

logtz_bay: BaYaka fathers’ log-transformed testosterone following z-score transformation

logtz_all: BaYaka and Bondongo fathers’ log-transformed testosterone following z-score transformation

provider: fathers’ raw Provider rankings; values reflect within-culture rankings from peers

providerz_bay: BaYaka fathers’ Provider rankings following z-score transformation

providerz_bon: Bondongo fathers’ Provider rankings following z-score transformation

providerz_all: an aggregate variable of “providerz_bay" and “providerz_bon”

dispute: fathers’ raw Dispute rankings; values reflect within-culture rankings from peers

disputez_bay: BaYaka fathers’ Dispute rankings following z-score transformation

disputez_bon: Bondongo fathers’ Dispute rankings following z-score transformation

disputez_all: an aggregate variable of “disputez_bay " and “disputez_bon”

share: BaYaka fathers’ raw Share rankings; values reflect within-culture rankings from peers

sharerz_bay: BaYaka fathers’ Share rankings following z-score transformation

teach: BaYaka fathers’ raw Teach rankings; values reflect within-culture rankings from peers

teachz_bay: BaYaka fathers’ Teach rankings following z-score transformation

[continues on next page]

**Notes on synthetic data for men’s ages and number of dependents**

Because the BaYaka and Bondongo communities are small, men’s ages and their number of dependents represent identifiable information. Therefore, we took two steps to help protect their identities and privacy. First, we used a published R script for a package known as “synthpop” to generate synthetic data for age and number of dependents that approximate the real data [97]. We then converted those two synthetic variables to z scores. Thus, we note that if one uses the provided data to replicate the analyses, the coefficients for the key predictors (i.e. fathers’ rankings) will be nearly identical, while the coefficients for age and number of dependents will differ, reflecting those synthetic variables being in standard scores.

97. Quintana, D. S. A synthetic dataset primer for the biobehavioural sciences to promote reproducibility and hypothesis generation. *Elife* **9**, e53275 (2020).

**Tables 1 and 2**

The .csv file is in a long file format. The analyses in Tables 1 and 2 reflect average values calculated after conversion of the file to a wide format.

Table 1: unpaired Student’s t-tests using “ethnicity” as the grouping variable.

Table 2: bivariate correlations for BaYaka men using Spearman’s *Rho.* We reported a small number of similar bivariate correlations (*Rho*) for Bondongo men.

**Tables 3 and 4**

The analyses in Tables 3 and 4 are OLS linear regression models with the standard errors clustered by “id” to account for repeated observations across individuals. In Stata v. 14.0, we conducted these analyses using the “reg” command followed by “vce(cluster id).”

Table 3: Four OLS regression models for each of the culturally-valued domains of fatherhood for BaYaka predicting “logtz_bay” as the dependent variable. We used the z-scores of each of the four fathering rankings as the key predictor in each relevant model.

Table 4: OLS regression models testing whether “ethnicity” moderated the relationship between Provider and Dispute (respectively) in predicting testosterone (“logtz_all”). Because there was no meaningful moderation effect for Dispute, we ran an additional model without an interaction term. We used the aggregated z-scores for Provider and Dispute rankings as the key predictors in each relevant model.
