# Supplementary material for: Sharing and caring: Testosterone, fathering, and generosity among BaYaka foragers of the Congo Basin
Source: Sci Rep. 2020 Sep 22;10:15422. doi: 10.1038/s41598-020-70958-3 (PMC7508877; doi:10.1038/s41598-020-70958-3)
Supplement: Supplementary file 3 — Supplementary information 3 [file 41598_2020_70958_MOESM3_ESM.docx]

Résumé français

Les humains, rares parmi les mammifères, font souvent preuve de soins paternels et d'une grande capacité d'hyper-coopération qui ont probablement été essentiels à l'émergence évolutive de l’histoire de vie humaine. Chez les humains et d’autres espèces, la testostérone est souvent un médiateur dans les compromis de l’histoire de vie humaine entre l'accouplement/compétition et le parentage. Il est également prouvé que les hommes ayant un taux de testostérone faible peuvent souvent avoir un comportement prosocial plus important que ceux avec un taux élevé. Étant donné l'importance évolutive des soins paternels et de la coopération accrue dans l’histoire de vie humaine, la testostérone des pères humains peut être liée à ces deux domaines comportementaux, mais ils n'ont pas été étudiés conjointement. Nous avons mené des recherches auprès de chasseurs-cueilleurs BaYaka congolais très égalitaires que nous avons comparés à leurs voisins pêcheurs-agriculteurs Bondongo à la structure sociale plus hiérarchisée. Nous avons évalué le niveau de testostérone des hommes BaYaka pour voir s’il était lié aux rôles paternels valorisés localement. Les résultats indiquent que les pères considérés comme les meilleurs partageurs dans la communauté avaient un taux de testostérone plus faible que ceux qui étaient moins généreux. Les pères BaYaka qui étaient de meilleurs pourvoyeurs avaient également tendance à un taux de testostérone plus faible. Les hommes dont le mariage était plus conflictuel avaient un taux de testostérone plus élevé et ce, tant chez les BaYaka et les Bondongo. Les résultats pour les pères BaYaka indiquent que la testostérone est un corrélat psychobiologique du comportement coopératif dans des conditions écologiques présentant des traits pertinents au plan évolutif, dans lesquelles l'aide mutuelle et le partage des ressources aident à assurer la survie et la santé de la communauté.
